# Pesticides for Apicultural and/or Agricultural Application Found in Belgian Honey Bee Wax Combs

**DOI:** 10.1007/s00128-015-1511-y

**Published:** 2015-03-07

**Authors:** Jorgen Ravoet, Wim Reybroeck, Dirk C. de Graaf

**Affiliations:** 1 Laboratory of Molecular Entomology and Bee Pathology, Ghent University, Krijgslaan 281 S2, 9000 Ghent, Belgium; 2 Technology and Food Science Unit, Institute for Agricultural and Fisheries Research (ILVO), Brusselsesteenweg 370, 9090 Melle, Belgium

**Keywords:** Wax combs, Beeswax, Pesticides, Honey bees, Residues

## Abstract

In a Belgian pilot study honey bee wax combs from ten hives were analyzed on the presence of almost 300 organochlorine and organophosphorous compounds by LC–MS/MS and GC–MS/MS. Traces of 18 pesticides were found and not a single sample was free of residues. The number of residues found per sample ranged from 3 to 13, and the pesticides found could be categorized as (1) pesticides for solely apicultural (veterinary) application, (2) pesticides for solely agricultural (crop protection) application, (3) pesticides for mixed agricultural and apicultural (veterinary) application. The frequencies and quantities of some environmental pollutants bear us high concerns. Most alarming was the detection of lindane (gamma-HCH) and dichlorodiphenyltrichloroethane (including its breakdown product dichlorodiphenyldichloroethylene), two insecticides that are banned in Europe. The present comprehensive residue analysis, however, also reveals residues of pesticides never found in beeswax before, i.e. DEET, propargite and bromophos.

Honey bees are at risk to be exposed to a broad set of chemicals originating from their environment and from beekeeping practices. Agricultural activities can be a source of a variety of pesticides. But the set of chemicals that are deliberately administered to the bee colonies by the beekeeper are at least equally impressive. Indeed, bee hives are treated against the ectoparasitic mite *Varroa destructor* with synthetic chemicals, like pyrethroid and organophosphate acaricides, naturally occurring organic acids and essential oils (Rosenkranz et al. 2010). Further, different antibiotics are commonly used in some countries for preventive or therapeutic treatment against American and European foulbrood, and against nosemosis (Reybroeck et al. 2012).

Nevertheless, there is a stringent legislation to prevent that these chemicals can pollute honey and thus come into the food chain. Authorization of these chemicals prohibit that farmers spray chemicals when crops are blooming. Besides, most disease control strategies should be applied after honey is yielded from the bee colonies. The honey is considered unfit for human consumption if residues are surpassing the maximum residue level (MRL) [Regulation (EC) No 396/2005]. Although the contamination of honey is widely documented in the scientific literature (reviewed by Bogdanov 2006; Wallner 1999), regular inspection of the quality of both the domestic and foreign honey by the Belgian authorities is rather reassuringly for agrochemicals and acaricides. Regarding antimicrobials the situation is somewhat different, mainly due to the absence of an MRL [Commission Regulation (EU) No 37/2010] for residues of antibiotics and sulphonamides in honey (Reybroeck et al. 2012).

Data on the contamination of other hive products like beeswax are much rarer and mostly come from nationwide surveys, rather than from regular inspections, simply because wax is in general considered as not consumed. On the other side, honey makes contact with the wax, comb honey is commercialized and beeswax is also used in some medicines and cosmetics. Earlier studies have shown the occurrence of mainly residues of the acaricides bromopropylate, coumaphos, amitraz and fluvalinate in beeswax (Chauzat and Faucon 2007; Mullin et al. 2010). Although acaricide residues in beeswax can affect the mite survival, depending on the type, concentration and presence of a honey bee cocoon (Fries et al. 1998), they are also likely to implicate acaricide resistance (Rosenkranz et al. 2010). Studying beeswax is particularly interesting because most of the pesticides and acaricides are fat soluble, non-volatile and persistent, and so accumulate easily herein. Moreover, these chemicals resist the wax melting temperature. Therefore they can accumulate for decades as it is a common beekeeping practice to recycle wax almost continuously in the form of foundations on which bees will construct a complete comb.

In the present pilot study, a comprehensive residue screening of honey bee comb wax was performed in order to determine the type of pesticide contamination that occurs in our region (Belgium, Flanders). It should allow us to narrow the scope of future nationwide surveys.

## Materials and Methods

During hive control in spring 2012, wax combs were sampled from ten honey bee hives at apiaries located in Flanders at Heist-op-den-Berg (six samples), Leuven (three samples) and Ghent (one sample). As at least 100 g beeswax was required for the multiresidue analysis, we took two empty wax combs from each hive. The sampled bee colonies seemed healthy, with no clinical signs of infectious diseases or acute intoxication.

The beeswax was analyzed on the presence of 293 organochlorine and organophosphorous compounds at Intertek Food Services GmbH (Bremen, Germany) according to the European EN 15662 method (CEN 2008). The pesticide residues were determined by LC–MS/MS and GC–MS/MS following acetonitrile extraction/partitioning and clean-up by dispersive SPE-QuEChERS method. The limit of quantification (LOQ) is 10 µg/kg for most pesticides except for fenthion, iprovalicarb and pirimicarb (LOQ ranging from 20 to 70 µg/kg). For samples with residues of pesticides with a concentration above the limit of detection (LOD) but below the LOQ the laboratory provided us an estimation of the concentration.

The LC–MS/MS procedure enables to determine the following agents: Abamectin; Acetamiprid; Aldicarb; Aldicarb sulfone; Aldicarb sulfoxide; Amitraz; Azoxystrobin; Benalaxyl; Bitertanol; Boscalid; Bromacil; Bromuconazole; Bupirimate; Buprofezin; Cadusafos; Carbaryl; Carbendazim; Carbofuran; Carbofuran (3-Hydroxy-); Chloroxuron; Clofentezine; Clomazone; Clothianidin; Cymiazole; Cyproconazole; Cyprodinil; Demeton-S-methyl; Demeton-S-methyl; Diethofencarb; Diethyltoluamid (DEET); Difenoconazole; Diflubenzuron; Dimethoate; Dimethomorph; Dimoxystrobin; Diniconazole; Diphenylamine; Disulfoton; Disulfoton-PS-sulfone; Disulfoton-PS-sulfoxide; Ditalimfos; Diuron; Dodine; EPN; Epoxiconazole; Ethiofencarb; Ethoprophos; Ethoxyquin; Famoxadone; Fenamiphos; Fenarimol; Fenazaquin; Fenbuconazole; Fenhexamid; Fenoxycarb; Fenpropimorph; Fenpyroximate; Fenthion; Fenthion-oxon; Fenthion-PO-sulfone; Fenthion-PS-sulfone; Fenthion-sulfoxide; Fluazifop-P-butyl; Fluazinam; Fludioxonil; Flufenoxuron; Fluquinconazole; Flusilazole; Fonofos; Hexaconazole; Hexythiazox; Imazalil; Imidacloprid; Indoxacarb; Iprovalicarb; Isofenphos; Isofenphos-methyl; Isoproturon; Kresoxim-methyl; Linuron; Lufenuron; Malaoxon; Malathion; Mecarbam; Mepanipyrim; Mepronil; Metalaxyl; Metamitron; Metazachlor; Methiocarb; Methiocarb; Methiocarb; Methomyl; Methoxyfenozide; Metobromuron; Metolcarb; Metribuzin; Monolinuron; Myclobutanil; Nitenpyram; Nuarimol; Omethoate; Oxadixyl; Oxamyl; Oxydemeton-methyl; Penconazole; Pencycuron; Pirimicarb; Pirimicarb (Desmethyl-); Prochloraz; Propamocarb; Propargite; Propiconazole; Propoxur; Propyzamide; Pymetrozine; Pyraclostrobin; Pyridaben; Pyridaphenthion; Pyrifenox; Pyrimethanil; Pyriproxyfen; Quinoxyfen; Rotenone; Spinosad; Spirodiclofen; Spiromesifen; Spiroxamine; Tebuconazole; Tebufenozide; Tebufenpyrad; Teflubenzuron; Terbutylazine; Tetraconazole; Thiabendazole; Thiacloprid; Thiametoxam; Thiodicarb; Thiophanate-methyl; Triadimefon; Triadimenol; Trichlorfon; Trifloxystrobin; Triflumizole; Triforine.

The GC–MS/MS procedure enables to determine the following agents: Aclonifen; Acrinathin; Alachlor; Aldrin; Benfluralin; Bifenthrin; Binapacryl; Bromophos (-methyl); Bromophos-ethyl; Bromopropylate; Captan; Carbophenothion; Chlordane (alpha-, cis-); Chlordane (Oxy); Chlordane (gamma-, trans-); Chlorfenapyr; Chlorfenson; Chlormephos; Chlorobenzilate; Chloroneb; Chloropropylate; Chlorothalonil; Chlorpropham; Chlorpyrifos (-ethyl); Chlorpyrifos-methyl; Chlorthal-dimethyl; Chlorthion; Chlorthiophos; Chlozolinate; Coumaphos; Cyanofenphos; Cyanophos; Cyfluthrin; Cyhalothrin (lambda-); Cypermethrin; DDD (o,p-); DDD (p,p-); DDE (o,p-); DDE (p,p-); DDT (o,p-); DDT (p,p-); Deltamethrin; Diazinon; Dichlobenil; Dichlofenthion; Dichlofluanid; Dicloran; Dicofol; Dieldrin; Endosulfan (alpha-); Endosulfan (beta-); Endosulfan-sulfat; Endrin; Esfenvalerate; Ethion; Etofenprox; Etridiazole; Etrimfos; Famphur; Fenchlorphos; Fenitrothion; Fenpropathrin; Fenson; Fensulfothion; Fenvalerate; Fluchloralin; Flucythrinate; Fluvalinate,; Folpet; Formothion; Halfenprox; HCH (alpha-); HCH (beta-); HCH (delta-); Heptachlor; Heptachlor epoxide (cis-, exo-); Heptachlor epoxide (trans-, endo-;); Heptenophos; Hexachlorobenzene (HCB); Hexaflumuron; Iodofenphos; Iprobenfos; Iprodione; Isazofos; Isocarbofos; Isodrin; Isoxathion; Leptophos; Lindane (gamma-HCH); Methacrifos; Methidathion; Methoxychlor; Mevinphos; Mirex; Monocrotophos; Nitrapyrin; Nitrofen; o-Phenylphenol; Paraoxon-ethyl; Paraoxon-methyl; Parathion-ethyl; Parathion-methyl; Pendimethalin; Pentachloroaniline; Pentachloroanisole; Permethrin; Phenthoate; Phorate; Phorate-sulfone; Phosalone; Phosmet; Phosphamidon; Piperonyl butoxide; Pirimiphos-ethyl; Pirimiphos-methyl; Procymidone; Profenofos; Profluralin; Propetamphos; Prothiophos; Pyrazophos; Quinalphos; Quintozene; S 421 (octachlorodipropyl ether); Sulfotep; Sulprofos; Tecnazene; Tefluthrin; Terbufos; Tetrachlorvinphos; Tetramethrin; Tetradifon; Tetrasul; Thionazin; Tolclofos-methyl; Tolylfluanid; Triallate; Triazophos; Trichloronat; Trifluralin; Vinclozolin.

Both procedures enables to determine the following agents: Acephate; Azinphos-ethyl; Azinphos-methyl; Chlorfenvinphos; Dichlorvos; Fipronil; Methamidophos.

## Results and Discussion

Traces of 18 pesticide residues were found in the beeswax samples and not a single sample was free from pesticide residues (Table 1). Of the in total 65 identifications 19 were below the LOQ (see Table 1: estimated concentrations are given between brackets) and 40 were above the MRL (MRL for residues in honey; see also comment here below). Moreover, the number of residues found per sample ranged from 3 to 13, and the pesticides found could be categorized as (1) pesticides for solely apicultural (veterinary) application, (2) pesticides for solely agricultural (crop protection) application, and (3) pesticides for mixed agricultural and apicultural (veterinary) application. The most frequently detected residues were fluvalinate (in all samples), coumaphos (in 90 % of the samples) and bromopropylate (in 70 % of the samples). For information of the reader, Table 1 also includes the MRL values for honey, which does not corresponds to the matrix that was analyzed in the present paper. Many of the pesticides detected are lipophilic and would likely remain in the wax rather than move to the honey that is stored in wax combs.

**Table 1:** Pesticides found in Belgian beeswax samples (n = 10)

Pesticide	Concentration (µg/kg)^f^	# Positive	Acute 48 h LD50 (µg/bee)	Type	MRL in honey (µg/kg)		Registration in Belgium		Earlier finding in beeswax
					Due to VMP use	Due to Pesticide use	VMP use in beekeeping	Pesticide use	
Pesticides used in beekeeping									
Coumaphos	(6; 7; 8); 15; 16; 31; 35; 39; 66	9	No data	aca	100	10	No^a^ (forbidden since 2009)	No	Yes
Pesticides used in beekeeping and/or applied to crops									
Bromopropylate	(7); 11; 18; 46; 46; 78; 89	7	183	aca	No MRL	10	No^a^ (forbidden since 2007)	No (forbidden since 2007)	Yes
Tau-fluvalinate	11; 12; 17; 17; 23; 27; 28; 30; 40; 83	10	No data	ins, aca	No MRL required	10	No^a^ (forbidden since 2008)	Yes	Yes
Amitraz	11; 19	2	50	ins, aca, vet	200	10	No^a^ (forbidden since 2006)	No (forbidden since 2004)	Yes
Piperonyl butoxide	10	1	294	opc, vet	No MRL required	10^g^	No	Yes^b^	Yes
Pesticides applied to crops									
Gamma-HCH	(7)	1	0.011	ins, aca	No MRL	10	No	No	Yes
Delta-HCH	(5; 8; 8; 9; 9); 13; 29	7	0.011^c^	ins, aca	No MRL	10	No	No	
Alpha-HCH	(9)	1	0.011^c^	ins, aca	No MRL	10	No	No	
DEET	13; 35; 36; 38; 41	5	No data	ins, rep	No MRL	10^g^	No	No^d^	
Propargite	12; 17; 27; 43; 65	5	47.9	aca	No MRL	10^g^	No	No	
Chlorfenvinphos	(8; 9); 11; 13; 15	5	0.55	ins, aca, vet	No MRL	10	No	No	Yes
p,p′-DDE	10	1	5^e^	ins	No MRL	50	No	No	Yes
p,p′-DDT	(6; 8); 44	3	5^e^	ins	No MRL	50	No	No	
o,p′-DDT	11	1	5^e^	ins	No MRL	50	No	No	
4,4′-Dibromobenzo Phenone	(5; 7; 8)	3	No data		No MRL	10^g^	No	No	Yes
Boscalid	12; 13	2	100	fun	No MRL	500	No	Yes	Yes
Parathion-methyl	16	1	19.5	ins	No MRL	10	No	No	Yes
Bromophos	(6)	1	0.44	ins	No MRL	10^g^	No	No	

Coumaphos : O,O-diethyl O-3-chloro-4-methyl-2-oxo-2H-chromen-7-yl phosphorothioate; p,p’-DDE: 1,1-bis-(4-chlorophenyl)-2,2-dichloroethene, p,p’-dichlorodiphenyldichloroethylene; o,p’-DDT: 1,1,1-trichloro-2-(2-chlorophenyl)-2-(4-chlorophenyl)ethane or o,p’-dichlorodiphenyltrichloroethane; p,p’-DDT: p,p’-dichlorodiphenyltrichloroethane; DEET: N,N-diethyl-meta-toluamide, diethyltoluamide, delphene or detamide; 4,4’-dibromobenzophenone: also known as bis(4-bromophenyl)methanone; HCH: hexachlorocyclohexane

*MRL* maximum residue limit/maximum residue level, *VMP* veterinary medicinal product, *aca* acaricide, *ins* insecticide, *vet* veterinary treatment, *rep* repellant, *fun* fungicide, *opc* other product constituent

^a^No longer registered to be used as a veterinary drug in beekeeping against *Varroa destructor*

^b^Not a plant protection product but a synergist

^c^Data for lindane = gamma-HCH

^d^No registration to be applied to crops, on the market as insect repellent

^e^Data for DDT

^f^Estimated concentration given between brackets for concentrations <LOQ, *LD50* lethal dose for 50 % of subjects

^g^Default value since no MRL was established

Fluvalinate is used for both agricultural and beekeeping purposes. In Belgium, it was a widely used acaricide in the first years after the *V. destructor* mite established in 1984. However, it was abandoned by most of the beekeepers once fluvalinate-resistant mites were found all over Europe. Besides, a veterinary medicinal product with fluvalinate as active substance is no longer registered for apicultural use in Belgium. The pesticide coumaphos is or was solely used in beekeeping and hence point to a beeswax contamination caused by apicultural practices. It is difficult to say whether it represent a recent or historic contamination. Bromopropylate was used in the early years of *Varroa*-treatments (commercialized as fumigation strips under the name Folbex VA) (Ritter and Perschil 1983), next to agricultural use, but it is currently banned in Europe (Commission Directive 2004/59/EC). Different authors also reported residues of bromopropylate in honey (Bogdanov 2006; Wallner 1999), which led to the discontinued use of Folbex VA. So, its detection in beeswax points towards a historic contamination.

Amitraz was found in two out of ten samples and could originate from both apicultural and agricultural applications in the past. Both applications are presently no longer allowed in Belgium. Since this acaricide was proven to be unstable in beeswax and honey (Jimenez et al. 1997; Korta et al. 2001), we presume that its presence denotes a recent *Varroa*-treatment with an amitraz-based product. This is remarkably as Apivar, the amitraz-based commercialized product for *Varroa*-treatment, is no more allowed in Belgium since 2006 when its authorization expired. The same is true for Apistan (based on fluvalinate; in Belgium no more allowed since 2008) and Perizin (based on coumaphos; in Belgium no more allowed since 2009). However, illegal sales of prescription drugs or the use of home-made preparations based on the agrochemical formulations cannot be excluded.

Piperonyl butoxide is registered for topical use in cows, sheep, goats and horses (Equidae) with no MRL required in food from these animals [Commission Regulation (EU) No 37/2010]. In theory piperonyl butoxide could be allowed to be used in beekeeping upon prescription of a veterinarian under ‘cascade’. The use of this pesticide in beekeeping cannot be excluded: it is sometimes used in insect and bee repellents which are on the market for use in beekeeping and some authors claim that piperonyl butoxide is enhancing the toxicity of fluvalinate to control *V. destructor* (Hillesheim et al. 1996). Piperonyl butoxide has the status of ‘not a plant protection product’ under Regulation (EC) No 1107/2009; but is a synergist. The provisions of Regulation (EC) No 396/2005 are not applicable.

All other found pesticides originate from a pollution of outside the beehive and their frequencies and quantities bear us high concerns. We have found several fungicides and crop protection agents that are highly toxic for honey bees (Table 1). All of them except boscalid are not or no longer authorized in the European Union for agricultural use by Regulation (EC) No 1107/2009. Two of them, lindane (gamma-HCH) and dichlorodiphenyltrichloroethane [DDT; including its breakdown product dichlorodiphenyldichloroethylene (DDE)] are even banned in Europe since several years. Both lindane and DDT are in fact mixtures of several isomers. Their occurrence in beeswax has been reported earlier (Chauzat and Faucon 2007; Mullin et al. 2010; Nguyen et al. 2009) and most probably points to a historic contamination and the failure to eliminate it completely due to the beekeeping practice of wax recycling. The present comprehensive residue analysis, however, also reveals residues of pesticides to our knowledge never found in beeswax before, i.e. DEET, propargite and bromophos.

Just like two other Belgian studies we failed to find traces of neonicotinoids in beeswax (Nguyen et al. 2009; Simon-Delso et al. 2014). Neonicotinoids are a class of neuro-active insecticides that are among the culprits of bee mortality, and the subject of debate in Europe and beyond. So far only few of the studies that were undertaken succeeded in determining their presence in beeswax (Mullin et al. 2010; Yanez et al. 2013), though these insecticides are frequently found in other matrices (honey bees, pollen, honey; Mullin et al. 2010) and can cause adverse effects at ppb-levels (Blacquière et al. 2012).

Worker bees of all ages are susceptible to the effects of pesticide exposure (Rortais et al. 2005), but beeswax contamination primarily affects the brood because of its direct contact with the brood cell wall. This developmental exposure to pesticides in brood combs will affect larval development (Wu et al. 2011) but also post-emergence fitness and performance of adult workers and queens (Wu et al. 2012; Pettis et al. 2004; Rangel 2013; Collins and Pettis 2013). Moreover, transfer from the beeswax matrix to the stored honey was experimentally demonstrated for pesticides (Wallner 1995) and sulfonamides (Reybroeck et al. 2010). Hence, even without any recent environmental exposure, newly deposited honey can become contaminated by historical pollution of the beeswax.

Given the contamination of beeswax one may consider to take actions in the field, aimed at lowering the pesticide contamination of beeswax in Belgian apiaries, as stated also before in other countries (Pettis et al. 2004).

## References

[CR1] Blacquière T, Smagghe G, van Gestel CAM, Mommaerts V (2012). Neonicotinoids in bees: a review on concentrations, side-effects and risk assessment. Ecotoxicology.

[CR2] Bogdanov S (2006). Contaminants of bee products. Apidologie.

[CR3] CEN (2008). European Standard EN 15662: foods of plant origin—determination of pesticide residues using GC–MS and/or LC–MS/MS following acetonitrile extraction/partitioning and clean-up by dispersive SPE—QuEChERS-method. Comité Européen de Normalisation

[CR4] Chauzat MP, Faucon JP (2007). Pesticide residues in beeswax samples collected from honey bee colonies (*Apis mellifera* L.) in France. Pest Manag Sci.

[CR5] Collins AM, Pettis JS (2013). Correlation of queen size and spermathecal contents and effects of miticide exposure during development. Apidologie.

[CR6] Fries I, Wallner K, Rosenkranz P (1998). Effects on *Varroa jacobsoni* from acaricides in beeswax. J Apic Res.

[CR7] Hillesheim E, Ritter W, Bassand D (1996). First data on resistance mechanisms of *Varroa jacobsoni* (Oud) against tau-fluvalinate. Exp Appl Acarol.

[CR8] Jimenez JJ, Bernal JL, delNozal MJ, Toribio L, Atienza J (1997). Characterization and monitoring of amitraz degradation products in honey. J High Resolut Chromatogr.

[CR9] Korta E, Bakkali A, Berrueta LA, Gallo B, Vicente F, Kilchenmann V, Bogdanov S (2001). Study of acaricide stability in honey. Characterization of amitraz degradation products in honey and beeswax. J Agric Food Chem.

[CR10] Mullin CA, Frazier M, Frazier JL, Ashcraft S, Simonds R, van Engelsdorp D, Pettis JS (2010). High levels of miticides and agrochemicals in North American apiaries: implications for honey bee health. PLoS One.

[CR11] Nguyen BK, Saegerman C, Pirard C, Mignon J, Widart J, Tuirionet B, Verheggen FJ, Berkvens D, De Pauw E, Haubruge E (2009). Does imidacloprid seed-treated maize have an impact on honey bee mortality?. J Econ Entomol.

[CR12] Pettis JS, Collins AM, Wilbanks R, Feldlaufer MF (2004). Effects of coumaphos on queen rearing in the honey bee, *Apis mellifera*. Apidologie.

[CR13] Rangel J (2013) The effects of miticides on the mating health of honey bee (*Apis mellifera* L.) queens. In: XXXXIII International Apicultural Congress, Apimondia (Kyiv), p 187

[CR14] Reybroeck W, Jacobs FJ, De Brabander HF, Daeseleire E (2010). Transfer of sulfamethazine from contaminated beeswax to honey. J Agric Food Chem.

[CR15] Reybroeck W, Daeseleire E, De Brabander HF, Herman L (2012). Antimicrobials in beekeeping. Vet Microbiol.

[CR16] Ritter W, Perschil F (1983). Determination of action of Folbex-Va (Isopropyl-4, 4′-Dibromobenzilate) against *Varroa* mites and of bee tolerance. Apidologie.

[CR17] Rortais A, Arnold G, Halm MP, Touffet-Briens F (2005). Modes of honeybees exposure to systemic insecticides: estimated amounts of contaminated pollen and nectar consumed by different categories of bees. Apidologie.

[CR18] Rosenkranz P, Aumeier P, Ziegelmann B (2010). Biology and control of *Varroa destructor*. J Invertebr Pathol.

[CR19] Simon-Delso N, San Martin G, Bruneau E, Minsart LA, Mouret C, Hautier L (2014). Honeybee colony disorder in crop areas: the role of pesticides and viruses. PLoS One.

[CR20] Wallner K (1995). The use of varroacides and their influence on the quality of bee products. Am Bee J.

[CR21] Wallner K (1999). Varroacides and their residues in bee products. Apidologie.

[CR22] Wu JY, Anelli CM, Sheppard WS (2011). Sub-lethal effects of pesticide residues in brood comb on worker honey bee (*Apis mellifera*) development and longevity. PLoS One.

[CR23] Wu JY, Smart MD, Anelli CM, Sheppard WS (2012). Honey bees (*Apis mellifera*) reared in brood combs containing high levels of pesticide residues exhibit increased susceptibility to *Nosema* (Microsporidia) infection. J Invertebr Pathol.

[CR24] Yanez KP, Bernal JL, Nozal MJ, Martin MT, Bernal J (2013). determination of seven neonicotinoid insecticides in beeswax by liquid chromatograph coupled to electrospray-mass spectrometry using a fused-core column. J Chrom A.

